# Phosphine Sorption on Paddy Rice: Effects on Fumigation and Grain Quality Parameters

**DOI:** 10.3390/foods13203293

**Published:** 2024-10-17

**Authors:** Silvia Andréia Garibaldi Pereira, Lázaro da Costa Corrêa Cañizares, Silvia Leticia Rivero Meza, Cristiano Dietrich Ferreira, Silvia Naiane Jappe, Gustavo Heinrich Lang, Paulo Carteri Coradi, Maurício de Oliveira

**Affiliations:** 1 Department of Agroindustry Science and Technology, Federal University of Pelotas, Pelotas 96010-900, RS, Brazil; silviagronomia@hotmail.com (S.A.G.P.); lazarocoosta@hotmail.com (L.d.C.C.C.); silvialrmeza@gmail.com (S.L.R.M.); jappesilvia@gmail.com (S.N.J.); gustavo.heinrich@hotmail.com (G.H.L.); mauricio@labgraos.com.br (M.d.O.); 2 Technological Institute in Food for Health, University of Vale do Rio dos Sinos, São Leopoldo 93022-750, RS, Brazil; cristiano.d.f@hotmail.com; 3 Laboratory of Postharvest (LAPOS), Campus Cachoeira do Sul, Federal University of Santa Maria, Avenue Taufik Germano, Universitário II, Cachoeira do Sul 96503-205, RS, Brazil

**Keywords:** phosphine sorption, phosphine residues, rehydration capacity, stored rice

## Abstract

During storage, infestation by insect pests occurs, causing quantitative and qualitative losses in grains, which requires the control of these insects with phosphine gas. Rice husk has a high phosphine adsorption capacity, influencing the gas concentration during fumigation and potentially leading to inefficient fumigation. Additionally, the high sorption of rice husk results in a higher residue of phosphine in the grain. Therefore, the objective of this study was to evaluate the phosphine sorption and phosphine residue in rice husk, paddy rice, and brown rice, as well as the industrial quality (head rice yield, rehydration capacity, cooking time, colorimetric profile) of brown and white rice during storage. To achieve this, fumigation of paddy rice, brown rice, and rice husks with 3.0 g·m^−3^ of phosphine was carried out for 240 h (recommended duration in the industry). A high sorption rate was observed in the rice husk (94.77%), paddy rice (97.61%), and, lastly, brown rice (35.17%). Due to the high sorption rate, only brown rice maintained a concentration above the recommended level for effective pest control (400 ppm for 120 h). Higher phosphine residues than permitted were observed in the rice husk (0.25 ppm). Lower rice head yields were observed in non-fumigated rice samples when analyzing the brown rice samples (66.21% for paddy rice and 65.01% for brown rice). A greater rehydration capacity was observed in fumigated samples at the beginning of storage when analyzing the brown rice samples (1.21 for paddy rice, 1.23 for brown rice), reducing the cooking time (24.00 for paddy rice, 23.80 for brown rice). More studies should be carried out to evaluate the effectiveness of fumigation on paddy rice, considering the high sorption rate of the paddy.

## 1. Introduction

Rice (*Oryza sativa* L.) is the second most widely produced cereal globally and is renowned for its high concentration of starch, proteins, minerals, and B-complex vitamins, making it a vital source of energy [1,2]. This grain serves as a staple food for half of the world’s population and is consumed by 95% of people worldwide [3].

After harvesting, the grains are transported to processing facilities, where they undergo cleaning and drying to achieve an appropriate moisture level for maintaining the grain quality during storage [4]. The grains are then stored in metal silos or warehouses to ensure a year-round supply of the product [5]. However, during storage, the proliferation of insects and deteriorating microorganisms can occur, leading to quantitative and qualitative losses. Phosphine (PH3), a highly toxic gas, is the primary method for insect control during grain storage, as it rapidly inhibits aerobic respiration in many insects [6,7].

The residue from aluminum phosphide application is a light gray powder composed of aluminum hydroxide. Aluminum hydroxide is an inert substance that is non-toxic to humans and animals, but it may contain undecomposed aluminum phosphide particles impregnated in the powder [8]. The maximum residue limits of aluminum phosphide allowed in samples after fumigation are 0.1 ppm or 0.1 mg/kg for grains [9].

During phosphine application, the gas concentration inside the environment decreases, primarily due to sorption by the grains, as well as other factors such as gas loss through the structure. Sorption is influenced by various factors, including the type of fumigated product, previous fumigation history, moisture content, particle size, composition, exposure time, and dosage [10]. Rice husk, which constitutes 20% of the grain weight, has a high silica content and specific surface area (10,197.38 cm^2^ g^−1^), leading to a high sorption rate of phosphine compared to other grains [11].

Phosphine gas is known to affect various metabolic processes in rice grains [12]. Niu et al. [7] analyzed the physiological and biochemical responses of rice seeds to phosphine exposure during germination. They observed a sudden decrease in the germination rate, germination potential, and germination index after exposure to phosphine. Additionally, seed exposure to phosphine reduced the activities of the catalase and superoxide dismutase enzymes while increasing the peroxidase activity compared to the control group.

No reports have yet addressed the effect of aluminum phosphide application on the phosphine sorption by rice husk and its influence on quality parameters during storage. Therefore, this study aimed to assess phosphine sorption and phosphine residue in rice husk, paddy rice, and brown rice, along with the industrial quality aspects (head rice yield, rehydration capacity, cooking time, colorimetric profile) of brown and white rice during storage.

## 2. Materials and Methods

### 2.1. Materials and Experimental Procedure

The rice was obtained from Cooperativa Arrozeira Extremo Sul (Pelotas, RS, Brazil). The long rice variety (Indica) was used. After harvesting, the samples were transported to the Laboratório de Pós-Colheita, Industrialização e Qualidade de Grãos (LABGRÃOS), of the Federal University of Pelotas (UFPel) (Latitude 31°52′00″ S; Longitude 52°21′24″ W; Altitude 13.24 m). The rice was cleaned and dried at 35 °C with an airflow of 1.0 m s^−1^ in an experimental fixed-bed dryer until a 12% moisture content was reached. Three samples were used for the experiment: paddy rice, brown rice, and rice husks.

**Figure 1:**
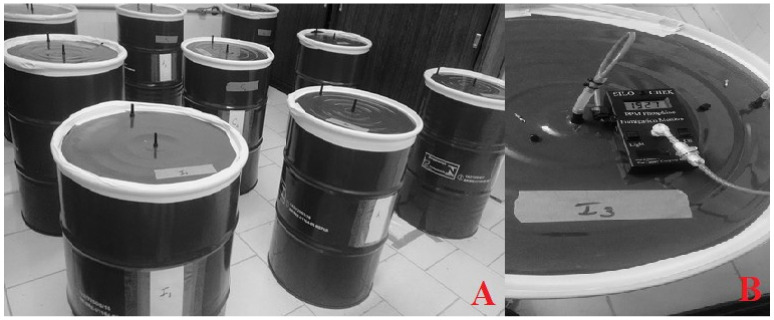
Metallic drums used for fumigation (**A**) and Silo-Chek—Canary Co device (**B**).

The samples were placed in metal drums at 28 °C and 55% relative humidity (Climate-controlled rooms with temperature and relative humidity control—Appropriate conditions for fumigation), hermetically sealed, with a capacity of 200 L and 3.0 g·m^−3^ of phosphine for 240 h (recommended time in the industry) (Figure 1A). The fumigation of each treatment was performed in triplicate (biological replicates).

After applying the treatments, the samples were stored in plastic bags (polyethylene) in a BOD incubator (ELETROlab—EL222/3) with temperature (25 °C) and humidity (60%) control for 3 months. Analysis was performed at the beginning of storage and at 1, 2, and 3 months. Analyses were performed on brown rice samples and after polishing (white rice). Storage of paddy and brown rice was also carried out without fumigation. The laboratory analyses were performed in triplicate (analytical replicates).

### 2.2. Methods

#### 2.2.1. Phosphine Concentration

The phosphine concentration was monitored using the Silo-Chek—Canary Co device (Canary Company Pty Ltd., Sydney, Australia) (Figure 1B). The phosphine concentration was measured every hour during the first day and every 12 h during the subsequent 9 days. To determine the percentage of phosphine sorption, the following equation was used:

Sorption (%) = [(A − B)/A] × 100,(1)

where “A” is the higher concentration (ppm) of phosphine during fumigation, and “B” is the lower concentration (ppm) of phosphine during fumigation.

#### 2.2.2. Phosphine Residue

This analysis aims to quantify the concentration of phosphine present in the grains after fumigation (phosphine residue). The quantification was performed using HPLC according to Analytical Methods for Pesticides Residues in Foodstuffs [13]. The analysis was performed on samples after 10 days of treatment. Along with this, the samples were aerated, and after 4 days of aeration, the samples were also collected to determine the residual phosphine concentration.

#### 2.2.3. Head Rice Yield (HRY)

For the quantification of the head rice yield, a Testing Rice Mill (DTAZ1, Zaccaria, Limeira, Brazil) was used. This equipment is employed for dehusking, polishing, and separating broken grains (<4.49 mm). The head rice yield (%) was determined by dividing the head rice weight (g) (brown and polished rice) (wf), with lengths higher than 4.49 mm [14], by the initial rice weight (g) (rice with husk) (wi). The results are expressed in % through the average of three determinations.

HRY (%) = (wf/wi) × 100,(2)

#### 2.2.4. Rehydration Capacity

The rehydration ratio of the rice samples was determined using the method described by Cao et al. [15]. The quantity of 5 g of rice was submerged in distilled water at 100 °C for 10 min in a hot plate (IKA C-MAG HS digital IKAMAG™, Staufen, Alemanha). The rehydration capacity is expressed as the ratio of wet weight to dry weight.

#### 2.2.5. Cooking Time

The cooking time of the rice was determined according to the method described by Juliano and Bechtel [16] and expressed as minutes (min). The quantity of 10 g of rice was placed in aluminum containers under heating with excess distilled water in a hot plate (IKA C-MAG HS digital IKAMAG™). The removal of 3 rice grains was performed at different time intervals. The removed grains were pressed between glass plates. The cooking time was determined when the pressed rice showed no white core.

#### 2.2.6. Colorimetric Profile

The colorimetric profile of the grains was determined using a colorimeter (Minolta, CR-310, Osaka, Japan). Through the CIELab system, the parameters used are the a*-value (positive = red and negative = green), b*-value (positive = yellow and negative = blue), and L*-value (100 = white and 0 = black). The rice grains were dispersed on Petri dishes, and 10 readings were taken for each sample.

#### 2.2.7. Statistical Analysis

The experiment was conducted with a completely randomized design (CRD), with three replications (biological replicates). The analyses were performed in triplicates (analytical replicates), and these were subjected to an analysis of variance (ANOVA) with 95% reliability, using the SAS Viya Analytics Software program (SAS, Cary, NC, USA, https://www.sas.com/en_us/software/viya.html). When the independent variables (rice samples and storage time) presented significant effects, dismemberment was performed in simple effects. The comparison between the rice samples and storage time was carried out using Tukey’s test.

## 3. Results and Discussion

### 3.1. Phosphine Concentration

**Figure 2:**
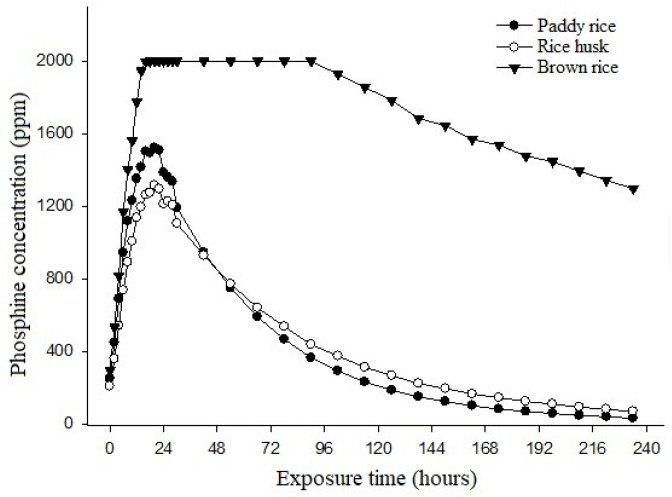
Phosphine concentrations during fumigation.

The results of the phosphine concentration in paddy rice, brown rice, and rice husk are presented in Figure 2. During the initial 20 h, an increase in phosphine concentration was observed. The maximum concentrations reached were 1520 ppm (20 h) for rice with husk, 2000 ppm (18 h) for brown rice, and 1314 ppm (20 h) for rice husk. The initial rise in the phosphine concentration is attributed to the reaction of the phosphate with the humidity that was present in the environment, releasing the phosphine gas [8]. The high concentrations persisted for a certain period, depending on the fumigated product, fumigation temperature and humidity, and the hermeticity of the fumigated environment [17].

In 4 h of fumigation for brown rice and paddy rice and 6 h for the husk, a minimum concentration of 400 ppm of phosphine was observed (Figure 2). Concentrations of 400 ppm were maintained for 77 h in paddy rice, 88 h in rice husks, and 236 h for brown rice. After this period of time, the phosphine concentrations tend to decrease to safe levels for sample removal. According to Lorini et al. [17], the minimum concentration for efficient control of all stages of insects (egg, larva, pupa, adult) is 400 ppm for 120 h. If the exposure time is less than 120 h, it may lead to inefficient insect control and selection of resistant insects. Thus, it can be seen that the paddy rice was not exposed to the minimum necessary period for adequate insect control to occur, which could lead to difficult maintenance of its quality during storage and/or processing.

The concentration of phosphine during fumigation is influenced by various factors, including the water activity, exposure time, and material being fumigated [18]. The lower concentration of phosphine observed during fumigation of paddy rice is attributed to the high sorption rate of rice husks, which constitutes 20% of the grain’s weight and has a high silica content [19].

After 240 h of fumigation, phosphine concentrations of 31.33, 68.67, and 1296.67 ppm were observed when analyzing the paddy rice, rice husks, and brown rice (Figure 2). Using Equation (2), the sorption of paddy rice was 97.61%, for rice husk, it was 94.77%, and for brown rice, it was 35.17%. These results suggest that the use of 3.0 g·m^−3^ of phosphine is not adequate for the efficient control of insects in stored rice grains due to the high sorption of phosphine by the rice husk, reducing its concentration in the environment. Further studies should be conducted to observe the influence of higher concentrations of phosphine during fumigation.

### 3.2. Phosphine Residue

**Figure 3:**
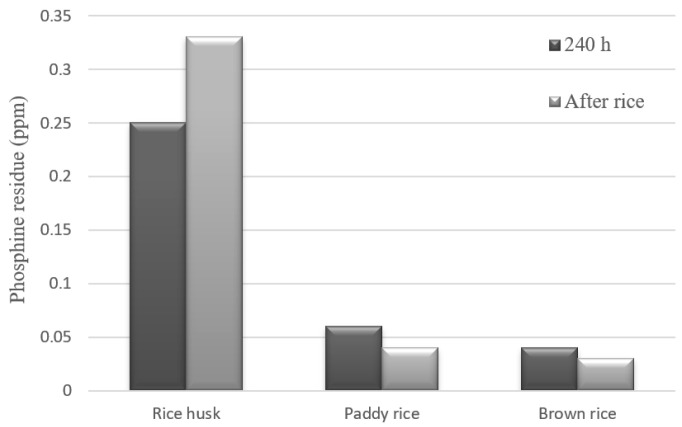
Phosphine residues after fumigation.

The results of phosphine residue in the paddy rice, brown rice, and rice husks are depicted in Figure 3. Initially, phosphine residues were analyzed in samples without fumigation, where no phosphine levels were detected. After 240 h of fumigation, phosphine levels of 0.25 ppm were observed in the rice husk, 0.06 ppm in the paddy rice, and 0.04 ppm in the brown rice. Following the fumigation period (240 h), the grains were aerated, and a residue analysis was conducted again after 4 days. The analysis revealed values of 0.33 ppm for rice husks, 0.04 ppm for paddy rice, and 0.03 ppm for brown rice.

The phosphine residue results correlate with previous phosphine sorption results, indicating that high levels of phosphine remain absorbed in the rice husk after fumigation, even with aeration. These residue values for rice husks exceed the limits allowed by Brazilian legislation (0.1 ppm). However, for the samples of paddy rice and brown rice, the results are within the limits (<0.1 ppm) set by the legislation. According to Daglish and Pavic [20], the rate of phosphine sorption is directly influenced by the moisture content and temperature during fumigation. Low concentrations of phosphine in the fumigated environment may indicate a high sorption rate by the grains.

### 3.3. Head Rice Yield (HRY)

The head rice yield results are summarized in Table 1. The analysis of variance indicated significant effects (*p* < 0.05) of the rice sample and storage time on the head rice yield. A declining trend in head rice yield was noted during storage for both the brown and white rice samples.

At the onset of storage, no significant differences were noted when comparing the rice samples. However, by the conclusion of the storage period, the lowest head rice yield was observed in the non-fumigated rice samples (paddy rice—not fumigated and brown rice—not fumigated).

Comparing the expurgated brown rice to the fumigated samples, higher levels of whole grains are evident. In the case of fumigated paddy rice, lower yield results were obtained, confirming the inefficiency of purging, which leads to higher contamination and degradation of grains during storage. No studies were found regarding the adsorption of rice husks concerning the fumigation efficiency and the yield of whole grains. When rice grains are stored under inadequate conditions (such as insect infestation, fungal infestation, high temperatures, and humidity), their constituents degrade, leading to grain damage and cracking. These actions result in increased broken grains during rice processing [21].

### 3.4. Rehydration Capacity and Cooking Time

The results for the rehydration capacity and cooking time of brown and white rice during storage are outlined in Table 2. The analysis of variance revealed significant effects (*p* < 0.05) of the rice sample on both rehydration capacity and cooking time. A decrease in rehydration capacity was noted during storage for white rice. In the case of brown rice samples, this reduction was observed only in the purged samples (paddy—fumigated and brown rice—fumigated).

Comparing fumigated and non-fumigated rice samples at the beginning of storage reveals that fumigation led to an increase in rehydration capacity for brown rice (1.21 for paddy rice—fumigated and 1.23 for brown rice—fumigated). This increase may be attributed to modifications in the grain structure upon contact with phosphine. When the phosphine penetrates the grain, it can react with hydrogen ions, lipids, and starch–protein interactions. These changes can increase the grain’s porosity, altering its physico-chemical properties and facilitating water absorption during cooking [22]. However, this phenomenon does not persist during storage. It is possible that over prolonged storage, bond restructuring occurs due to the reduction in residual phosphine present in the samples (Figure 3).

When analyzing polished rice samples (white rice), no significant differences were observed between fumigated and non-fumigated samples. This lack of difference is likely due to the removal of the bran, which contains a high protein concentration. Consequently, the reactions of phosphine with the bonds between starch and protein do not occur in polished rice.

The increase in rehydration capacity observed in the fumigated samples led to a reduction in cooking time for brown rice when analyzing the samples at the beginning of storage (24.00 min in paddy rice—fumigated and 23.80 min in brown rice—fumigated). This acceleration in cooking time is attributed to the rapid water absorption caused by the alteration in the structure of fumigated grains, which accelerates the gelatinization process of starch granules [23]. However, when analyzing polished samples (white rice), no significant differences were observed in cooking time, which aligns with the findings for rehydration capacity. Studies correlating fumigation with phosphine to cooking time and the rehydration capacity of rice grains were not found.

### 3.5. Colorimetric Profile

The colorimetric profile results for brown and white rice during storage are detailed in Table 3. The analysis of variance only indicated significant effects (*p* < 0.05) for the b* value when assessing white rice.

When analyzing the samples of brown rice, no significant differences were observed in the colorimetric profile, regardless of storage time. Similarly, for the samples of polished rice (white rice), no significant differences were found in the colorimetric profile at the beginning of storage. However, after 3 months of storage, the lowest b* value was observed in the paddy rice—fumigated (7.52) and brown rice—fumigated (7.57) samples. The increase in the b* value is associated with the yellowing of the grains, with higher b* values indicating more yellowish grains.

The increased yellowing in the non-fumigated rice during storage may be related to the incidence of deteriorating microorganisms, leading to the oxidation of the grain compounds. The incidence of microorganisms during storage occurs due to a lack of fumigation of the samples before storage. There are no reports in the literature regarding the effect of fumigation on the colorimetric profile.

## 4. Conclusions

This study analyzed the sorption of phosphine during the fumigation of different rice components such as rice husk, paddy rice, and brown rice. We found that rice husks exhibited the highest sorption rate, followed by paddy rice, with brown rice showing the lowest sorption rate. Due to this high sorption rate, only brown rice maintained phosphine concentrations above the recommended level for effective pest control. Excessive phosphine residues, exceeding permissible levels, were observed in the rice husks. Lower rice head yields were observed in non-fumigated rice samples, while a higher rehydration capacity was noted in the fumigated samples, leading to a reduced cooking time. Additionally, phosphine was found to cause oxidation and yellowing of polished (white) rice. In future studies, further investigations into the sorption behavior of phosphine during rice fumigation could lead to improved fumigation practices and the development of more efficient pest control strategies.

## Figures and Tables

**Table 1:** Head rice yield in brown rice and white rice, fumigated in the form of brown rice and paddy rice, during storage.

	Storage Time (Months)			
	0	1	2	3
	Head rice yield (%)—Brown rice			
Paddy rice (Fumigated)	69.54 ± 0.24 aA *	69.32 ± 0.12 aA	68.12 ± 0.21 bB	67.05 ± 0.32 bC
Paddy rice (Not fumigated)	70.23 ± 0.32 aA	69.01 ± 0.45 aB	67.21 ± 0.31 bC	66.21 ± 0.12 cD
Brown rice (Fumigated)	69.72 ± 0.82 aA	69.19 ± 0.45 aA	68.81 ± 0.07 aB	68.57 ± 0.41 aB
Brown rice (Not fumigated)	69.92 ± 0.76 aA	69.30 ± 0.31 aA	67.80 ± 0.11 bB	65.01 ± 0.19 cB
	Head rice yield (%)—White rice			
Paddy rice (Fumigated)	63.94 ± 0.74 aA	63.47 ± 0.41 aA	62.74 ± 0.08 bB	61.98 ± 0.31 bC
Paddy rice (Not fumigated)	64.66 ± 0.25 aA	63.88 ± 0.43 aA	62.12 ± 1.21 bB	61.08 ± 0.13 cC
Brown rice (Fumigated)	64.91 ± 1.11 aA	64.27 ± 0.27 aA	63.51 ± 0.18 aB	63.17 ± 0.19 aB
Brown rice (Not fumigated)	64.50 ± 0.67 aA	63.30 ± 0.20 aB	62.31 ± 0.09 bB	61.10 ± 0.09 cC

* The *p*-value for both rows and columns was *p* < 0.05. Lowercase letters compare between rice, uppercase letters compare between storage times.

**Table 2:** Rehydration capacity and cooking time in brown rice and white rice, fumigated in the form of brown rice and paddy rice, during storage.

	Storage Time (Months)			
	0	1	2	3
	Rehydration capacity—brown rice			
Paddy rice (Fumigated)	1.21 ± 0.01 aA *	1.17 ± 0.02 abB	1.12 ± 0.02 aB	1.10 ± 0.06 aB
Paddy rice (Not fumigated)	1.14 ± 0.01 bA	1.13 ± 0.05 bA	1.12 ± 0.01 aA	1.08 ± 0.03 aA
Brown rice (Fumigated)	1.23 ± 0.01 aA	1.18 ± 0.01 aB	1.13 ± 0.05 aB	1.12 ± 0.04 aB
Brown rice (Not fumigated)	1.14 ± 0.01 bA	1.14 ± 0.00 bA	1.13 ± 0.01 aA	1.10 ± 0.02 aA
	Rehydration capacity—white rice			
Paddy rice (Fumigated)	1.30 ± 0.01 aA	1.27 ± 0.01 aB	1.25 ± 0.00 aB	1.19 ± 0.03 aC
Paddy rice (Not fumigated)	1.27 ± 0.00 aA	1.27 ± 0.02 aB	1.25 ± 0.02 aB	1.15 ± 0.01 aC
Brown rice (Fumigated)	1.30 ± 0.01 aA	1.20 ± 0.01 aB	1.21 ± 0.01 aB	1.17 ± 0.01 aC
Brown rice (Not fumigated)	1.29 ± 0.03 aA	1.21 ± 0.02 aB	1.19 ± 0.03 aB	1.13 ± 0.01 aC
	Cooking time (min)—brown rice			
Paddy rice (Fumigated)	24.00 ± 0.00 bB	26.20 ± 0.10 aA	27.70 ± 0.70 aA	27.20 ± 0.50 aA
Paddy rice (Not fumigated)	26.50 ± 0.70 aA	26.30 ± 0.00 aA	28.70 ± 0.00 aA	27.10 ± 0.10 aA
Brown rice (Fumigated)	23.80 ± 0.00 bB	26.50 ± 0.70 aA	27.50 ± 0.00 aA	27.30 ± 0.20 aA
Brown rice (Not fumigated)	27.00 ± 1.40 aA	26.10 ± 0.00 aA	27.30 ± 0.00 aA	27.20 ± 0.10 aA
	Cooking time (min)—white rice			
Paddy rice (Fumigated)	14.22 ± 0.00 aB	15.10 ± 0.12 aA	15.25 ± 0.35 aA	15.10 ± 0.14 aA
Paddy rice (Not fumigated)	14.12 ± 0.00 aB	15.12 ± 0.05 aA	15.25 ± 0.35 aA	15.50 ± 0.10 aA
Brown rice (Fumigated)	14.15 ± 0.35 aB	15.22 ± 0.12 aA	14.75 ± 0.35 aA	15.20 ± 0.08 aA
Brown rice (Not fumigated)	14.10 ± 0.00 aB	15.50 ± 0.21 aA	15.50 ± 0.00 aA	15.15 ± 0.10 aA

* The *p*-value for both rows and columns was *p* < 0.05. Lowercase letters compare between rice, uppercase letters compare between storage times.

**Table 3:** Colorimetric profile of brown and white rice, fumigated in the form of brown rice and paddy rice, during storage.

	Storage Time (Months)			
	0	1	2	3
	Value L*—brown rice			
Paddy rice (Fumigated)	65.69 ± 2.22 aA *	65.10 ± 1.79 aA	65.08 ± 1.23 aA	64.90 ± 1.28 aA
Paddy rice (Not fumigated)	65.41 ± 1.83 aA	64.44 ± 1.94 aA	64.09 ± 1.48 aA	63.57 ± 1.13 aA
Brown rice (Fumigated)	65.54 ± 2.53 aA	65.22 ± 2.21 aA	64.96 ± 1.58 aA	64.78 ± 1.48 aA
Brown rice (Not fumigated)	65.53 ± 3.21 aA	64.41 ± 2.76 aA	63.97 ± 1.76 aA	63.51 ± 1.47 aA
	Value a*—brown rice			
Paddy rice (Fumigated)	1.14 ± 0.64 aA	1.78 ± 0.39 aA	1.78 ± 0.61 aA	1.48 ± 0.55 aA
Paddy rice (Not fumigated)	1.18 ± 0.60 aA	1.96 ± 0.24 aA	1.71 ± 0.30 aA	1.87 ± 0.28 aA
Brown rice (Fumigated)	1.64 ± 0.54 aA	1.86 ± 0.44 aA	1.39 ± 0.66 aA	1.79 ± 0.61 aA
Brown rice (Not fumigated)	1.43 ± 0.73 aA	1.62 ± 0.36 aA	1.47 ± 0.80 aA	1.52 ± 0.14 aA
	Value b*—brown rice			
Paddy rice (Fumigated)	20.53 ± 1.42 aA	18.97 ± 0.47 aA	18.39 ± 1.66 aA	18.27 ± 1.09 aA
Paddy rice (Not fumigated)	20.67 ± 1.61 aA	19.46 ± 0.50 aA	18.95 ± 1.19 aA	18.80 ± 0.61 aA
Brown rice (Fumigated)	20.42 ± 1.37 aA	19.74 ± 0.75 aA	18.54 ± 1.02 aA	18.30 ± 1.71 aA
Brown rice (Not fumigated)	20.95 ± 1.08 aA	19.79 ± 1.30 aA	18.52 ± 1.14 aA	18.75 ± 1.30 aA
	Value L*—white rice			
Paddy rice (Fumigated)	73.51 ± 1.39 aA	72.91 ± 0.77 aA	72.87 ± 0.35 aA	72.28 ± 0.69 aA
Paddy rice (Not fumigated)	73.09 ± 0.81 aA	72.22 ± 1.13 aA	72.43 ± 1.02 aA	71.06 ± 0.85 aA
Brown rice (Fumigated)	73.75 ± 0.83 aA	73.17 ± 0.51 aA	72.01 ± 1.02 aA	71.98 ± 0.52 aA
Brown rice (Not fumigated)	73.09 ± 1.01 aA	72.16 ± 0.96 aA	72.03 ± 0.78 aA	71.68 ± 0.67 aA
	Value a*—white rice			
Paddy rice (Fumigated)	−0.62 ± 0.15 aA	−0.67 ± 0.07 aA	−0.75 ± 0.13 aA	−0.87 ± 0.08 aA
Paddy rice (Not fumigated)	−0.68 ± 0.14 aA	−0.84 ± 0.10 aA	−0.89 ±0.11 aA	−0.97 ± 0.18 aA
Brown rice (Fumigated)	−0.52 ± 0.17 aA	−0.75 ± 0.15 aA	−0.95 ± 0.19 aA	−1.06 ± 0.11 aA
Brown rice (Not fumigated)	−0.65 ± 0.10 aA	−0.91 ± 0.14 aA	−0.95 ± 0.15 aA	−1.07 ± 0.18 aA
	Value b*—white rice			
Paddy rice (Fumigated)	8.38 ± 1.17 aA	8.28 ± 0.42 abA	8.15 ± 0.98 aA	7.52 ± 0.25 bA
Paddy rice (Not fumigated)	9.12 ± 1.07 aA	8.92 ± 0.52 bA	8.82 ± 0.55 aA	8.52 ± 0.32 aA
Brown rice (Fumigated)	8.42 ± 0.88 aA	8.31 ± 0.80 abA	8.28 ± 0.49 aA	7.57 ± 0.42 bA
Brown rice (Not fumigated)	9.72 ± 0.64 aA	8.64 ± 0.53 aA	8.56 ± 0.44 aA	8.45 ± 0.41 aA

* The *p*-value for both rows and columns was *p* < 0.05. Lowercase letters compare between rice, uppercase letters compare between storage times.

## Data Availability

No new data were created or analyzed in this study. Data sharing is not applicable to this article.
